# *That’s Cool*. Computational Sociolinguistic Methods for Investigating Individual Lexico-grammatical Variation

**DOI:** 10.3389/frai.2020.547531

**Published:** 2021-01-26

**Authors:** Hans-Jörg Schmid, Quirin Würschinger, Sebastian Fischer, Helmut Küchenhoff

**Affiliations:** ^1^ Department of English and American Studies, LMU, Munich, Germany; ^2^ Statistical Consulting Unit StaBLab, Department of Statistics, LMU, Munich, Germany

**Keywords:** individual variation, lexico-grammatical variation, social variation, corpus data, mixed-effects regression models, language change word count: 10, 380

## Abstract

The present study deals with variation in the use of lexico-grammatical patterns and emphasizes the need to embrace individual variation. Targeting the pattern that’s adj (as in *that’s right*, *that’s nice* or *that’s okay*) as a case study, we use a tailor-made Python script to systematically retrieve grammatical and semantic information about all instances of this construction in BNC2014 as well as sociolinguistic information enabling us to study social and individual lexico-grammatical variation among speakers who have used this pattern. The dataset amounts to 4,394 tokens produced by 445 speakers using 159 adjective types in 931 conversations. Using detailed descriptive statistics and mixed-effects regression models, we show that while the choice of some adjectives is partly determined by social variables, situational and especially individual variation is rampant overall. Adopting a cognitive-linguistic perspective and relying on the notion of entrenchment, we interpret these findings as reflecting individual speakers' routines. We argue that computational sociolinguistics is in an ideal position to contribute to the data-driven investigation of individual lexico-grammatical variation and encourage computational sociolinguists to grab this opportunity. For the routines of individual speakers ultimately both underlie and compromise systematic social variation and trigger and steer well-known types of language change including grammaticalization, pragmaticalization and change by invited inference.

## Introduction

Sociolinguistics, both “traditional” and computational, has focused on regionally, socially and situationally conditioned variation on the linguistic levels of phonology and morphosyntax. Deviating from this tradition, we investigate individual variation on the interface between lexis and grammar. Our main goal is to demonstrate that having a closer look at individual variation–rather than treating it as noise or residual variance–can contribute to a better understanding not only of regional and social variation but also of lexical, pragmatic and grammatical variation and language change.

Of course we are not the first to take a keen interest in individual variation in the use of linguistic features and patterns. In forensic linguistics and author identification studies (Coulthard 2004), individual differences regarding the use and frequency of linguistic patterns have taken center stage for some time. Milestone publications highlighting individual differences in the field of sociolinguistics include Guy (1980); Wolfram and Beckett (2000); Tagliamonte and Baayen (2012) and Walker and Meyerhoff (2013). However, the survey given by Walker and Meyerhoff (2013) shows two things: first, while many studies in variationist sociolinguistics, in fact starting as early as with Labov (1966), have acknowledged the importance of individual variation in principle, none of them have actually investigated the nature of individual variation and its implications in detail. And second, lexical or lexico-grammatical variation has not been addressed so far.

Individual differences regarding the mental representation of linguistic knowledge are the main concern of studies in the field of usage-based cognitive linguistics, for example by Barlow (2013) and Verhagen et al. (2018). In a similar vein, Dąbrowska (2015); Dabrowska (2016) has focused on individual differences in the language attainment of native and L2 speakers. Since individual speakers are the ultimate carriers of language change, it is not surprising that individual variation has been gaining increasing attention in corpus-based diachronic linguistics. Relevant publications include Gries and Hilpert (2010); Schmid and Mantlik (2015); Baxter and Croft (2016); Petré and Van de Velde (2018); Anthonissen (2020a); Anthonissen (2020b); Petré and Anthonissen (2020). Work in this tradition tends to be based on the assumption that frequency distributions in the works of individual authors can, under certain circumstances, be interpreted with regard to the writers’ underlying mental representations.

Taking insights from these fields into consideration, the present paper aims to encourage researchers in computational sociolinguistics to embrace the study of individual differences in lexico-grammatical variation. It is not our main intention to provide an in-depth investigation of the pattern under investigation, i.e., the pattern *that’s* Adj. Instead, we are using the pattern as an example to showcase potential methods to be extended in future work in computational sociolinguistics and to emphasize the relevance of studies of this type for understanding linguistic variation and change.

The paper is structured as follows. In *The Target Pattern:* *That’s Adj* we will describe the lexico-grammatical pattern chosen to serve as a target of the present case study, the pattern that's Adj as in *that’s right* or *that’s nice*. *Data* will report the computational methods developed to retrieve the kind of data required for the study of individual lexico-grammatical variation. *Results: descriptive data summary* will provide a descriptive statistical summary of the results regarding social, situational and individual variation. *Inferential statistics and results* will present the inferential-statistical techniques we have used to gauge the influence of social, situational and individual factors on the use of the pattern. *Discussion* will discuss the cognitive implications of our results and the role of individual variation vis-à-vis social variation and various types of language change.

## The Target Pattern: That’s Adj

The pattern investigated in this article is illustrated in examples (1) to (6), taken from BNC2014. Each example is related to one of six dominant usage types of the pattern.

(1)‘Evaluative’ use:
  S0255: er you just everything’s taken off you ev- totally everything’s taken er ou- off your hands.
  S0315: that’s fantastic (S28F)
(2)‘Epistemic’ use:
  S0519: in-interestingly the we are having the last two summers certainly worse summer because the Gulf Stream has shifted south.
  S0520: mm
  S0521: **that’s true** yeah (S24E)
(3)‘Ethical’ use:
  S0337: outside --ANONnameM’s grandad’s house you know there’s always cars there (.) someone was in like a Ford Focus and like maybe a Ford Fiesta and like er she clearly did n’t know how big her car was it was like full on not going anywhere and er would n’t go past a parked car.
  S0336: **that’s mean** (S985)
(4)‘Emotive’ use:
  S0585: yeah for er yeah exactly yeah and I was like ugh that is so horrible and she’s like yeah I threw up through my nose and I was like no
  S0587: that’s horrible (SNXG)
(5)‘Descriptive’ use:
  S0179: yeah (.) yeah (.) my opinion of him went down.
  S0058: that’s interesting (S37K)
(6)‘Discursive’ use:
  S0278: he’s a lovely fella ain’t he?
  S0013: course he is.
  S0278: well thank you very much.
  S0013: **that’s okay** (S7RA)

In all cases, the pattern that’s Adj is used in utterance-initial position or preceded by an interjection in this position. The demonstrative pronoun *that* refers to the content of one or more preceding utterances in what Halliday and Hasan (1976) call “extended anaphoric reference”. The predicate consists of the contracted form of the copula and an adjective. In all cases, the communicative goal motivating speakers to use this pattern is the wish to relate back to something mentioned in the previous cotext and express some sort of comment.

The examples given illustrate the six most common specific functions of the pattern. In (1) the speaker offers a positive evaluation, in (2) a comment on the truth or correctness of what was said, and in (3) an assessment from an ethical perspective. Example (4) has a predominantly emotive function, and (5) a descriptive one. In example (6) the pattern is mainly used to signal uptake of what was said by the previous speaker, i.e. it has a predominantly discursive function. It should be emphasized that these six functions are idealized peaks in what is in fact a rather scattered pragmatic and semantic landscape. One utterance can be motivated by several goals and express a combination of, say, evaluation, epistemic confirmation and discursive uptake. Since many adjectives, e.g. *right* or *fine* or *lovely*, can be chosen to realize different functions in one or different utterances, there is no one-to-one correspondence between adjective types and functions and meanings. Nevertheless, given the programmatic nature of the study, we will pretend that such a one-to-one correspondence actually exists and shoehorn each adjective into the functional category that it instantiates most typically and frequently as indicated by the data.

The pattern that’s Adj competes with a number of closely related patterns also offering the potential to combine extended anaphoric reference with various kinds of predications:

(7)THAT IS ADJ: e.g. *that is brilliant, that is interesting*
(8)THAT IS ADJ: e.g. *this is true, this is nice*
(9)IT’S ADJ: e.g. *it’s weird, it’s lovely*.
(10)THAT’S (A) N: e.g. *that’s nonsense, that’s a shame, that’s a lie*
(11)what a N: e.g. *what a shame, what a nightmare*

Even though these patterns clearly lie within the envelope of variation from an onomasiological perspective, they are not included in the present study. This restriction is necessary at this point to keep the methodological challenge within manageable bounds. Despite the fact that we are taking the form that’s Adj as our point of departure, we conceive of our investigation as a study in onomasiological variation, because we focus on how different communicative goals are encoded by the choice of different adjectives. Semasiological variation, i.e., variation in the meanings of specific forms such as *that’s right* or *that’s fine* lies outside the scope of this study but should be included in future work.

## Data

### Data Source

As the pattern under investigation is typically used in spontaneous spoken interaction, we decided to harvest data from the British National Corpus 2014 (BNC2014), which contains about 11 million words of transcribed casual conversations and has the additional advantage that metadata about speakers are available. BNC2014 is a successor to the British National Corpus (BNC).^1^ So far only the spoken component, collected between 2012 and 2016, has been published. All words have been tagged with regard to both part-of-speech and, remarkably, semantic information, using the Lancaster UCREL English semantic tagger (USAS).^2^ The corpus is also richly annotated with regard to various types of social and situational metadata.

### Data Retrieval

Data from the BNC2014 can be accessed and queried online using the CQPweb interface provided by Lancaster University^3^ or downloaded for use with individual processing methods. While the online platform offers a sophisticated interface to perform complex linguistic queries using the CQP query language, it does not enable users to include all metadata and to export results in a format that allows for more fine-grained analyses and filtering.

The freely available offline version of the BNC2014 provides all corpus texts with annotations in XML format as well as spreadsheets containing the full corpus metadata. Parsing this archive allows users to perform complex queries on the full textual data as well as to analyze hits according to conversation- and speaker-based information, which is essential for investigating variation in these two dimensions.

We therefore created a Python script that processes the BNC2014 data using XML parsing to enable queries based on all tags available in the textual data.^4^ In the case of the pattern that’s Adj we retrieved all utterances that either start with the pattern or where it is only preceded by interjections (pos: uh). Based on this restriction we collected all attestations that feature the singular determiner *that* (pos: dd1) followed by the item *‘s* and an adjective (pos: jj). In addition to collecting all instances of the pattern, the script outputs the total number of attestations in the corpus (n = 4,883).

An inspection of the results revealed a number of false positives, mainly stemming from tagging errors, which could be reduced by additional filtering using a blacklist of six tokens: *to, timing, news, bullshit, awesome, enough*.

For each hit we store the full attestation (e.g., *that’s good*), the slot-filling adjective (e.g., *good*), its semantic tag (e.g., A5:1) and its category description (e.g., Evaluation:- Good/bad), which we add from the USAS tagset. Besides, we record utterance, conversation and speaker IDs which allow us to automatically retrieve all metadata from the BNC2014 spreadsheets and to include it in the output: e.g., age, gender, birthplace of speakers or date, topic and type of conversations. Based on the list of speakers who have used the pattern we then query the full corpus to calculate the total number of words contributed by each individual, which is needed to determine normalized frequencies per speaker and to perform statistical tests targeting individual variation.

The Python script can be found in the supplementary material attached to this article. While this script has been tailored to detect instances of the that’s Adj pattern, it can be readily adapted to perform any XML-based query in the BNC2014 by modifying only the query part of the script.

### Manual Post-processing

Although the precision of the automatic processing was high, 268 false hits (amounting to 5.49%) had to be removed manually from the dataset. The major types of unwanted hits were: 1) uses of *that* which clearly functioned as relative rather than demonstrative pronouns (see 12); 2) uses of the pattern *that’s* Adj with deictic reference to objects accessible in the situational context (see 13); or 3) with anaphoric reference to antecedents referring to concrete objects (see 14).

(12)
  S0084: it’s better to find something that you can do.
  S0083: mm
  S0084: **that’s stable** in the short-term (.) and get a qualification that means you it will be stable rather than just here and there.
(13)
  S0245: what color do you want?
  S0246: grey
  S0245: I want grey shall we get two? it’s only two fifty (.) comes in bla-
  S0246: oh wait **that’s black** (BNC2014, S4QK 92).
(14)
  S0515: this is called the Lipstick Tower
  S0512: oh uhu
  S0515: **that’s modern** (BNC2014, SGAW 465).

The dataset had to be adjusted in three more ways. First, all 23 attestations contributed by three speakers in the age range 0–10 were removed. Also removed were 208 attestations that featured the value “unknown” for one or more social variable. And third, due to data scarcity in some of the age ranges, we re-categorized the variable age into five instead of the original 10 age ranges, comprising ages 11 to 18, 19 to 29, 30 to 49, 50 to 69 and 70 to 99, respectively.

### Final Dataset

The final dataset includes 4,394 attestations by 445 speakers in 931 conversations. These 4,394 tokens represent 159 adjective types. Boasting as many as 1,418 tokens, the most frequent adjective is *right*, followed by *good* (484 tokens) and *true* (340 tokens). 62 adjectives occur only once, 15 adjectives twice. The mean of tokens per adjective is 27.64, the median 3. The maximum number of tokens per speaker is 525, the minimum 1. The mean of tokens per speaker is 9.88, the median 3.

### Data Distribution for Major Social Variables

**FIGURE 1:**
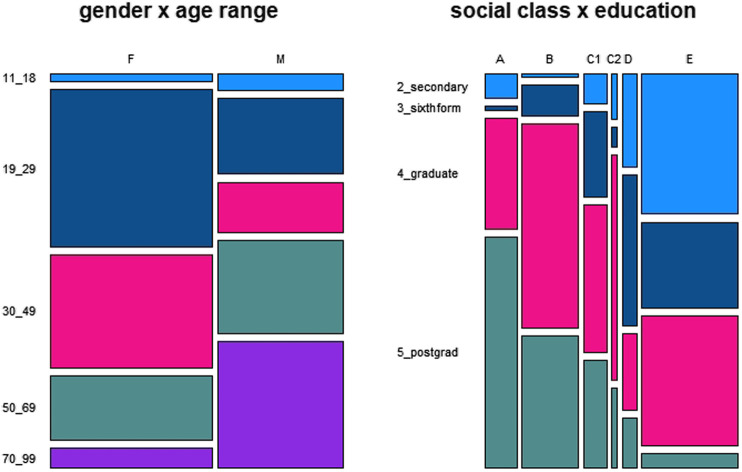
Data distribution across the variables gender and age and social class and education.

Generally, the data are not distributed evenly across the categories of the social variables included in the metadata of BNC2014. We focus on the distribution of the main variables gender, age, education and social class. As is indicated by the mosaic plot given in Figure 1, there are more data by women than by men, more data by young women than by older women and more data by older men than by young ones. As far as education and social class are concerned, Figure 1 indicates a substantial overrepresentation of social classes E and B and an expectable trend for a positive correlation between higher levels of education and social class.

## Results: Descriptive Data Summary

### Distribution of Tokens and Types Across Semantic Classes

As is shown in Table 1, there is no positive correlation between numbers of tokens and types. The class boasting the largest number of tokens, i.e., “epistemic”, is on the second-lowest rank regarding types, while “descriptive” is manifested by the largest number of types and the second-lowest number of tokens. The class “uptake” stands out because it is only represented by the three types *alright, fine* and *good* (see also Table 2).

**TABLE 1:** Distribution of tokens and types across semantic classes.

Class	Tokens	Types of adjectives
Epistemic	1853	9
Evaluative	1,177	33
Uptake	597	3
Emotive	472	37
Descriptive	468	63
Ethical	48	20
Total	4,615	165

**TABLE 2:** Most frequent adjectives per semantic class.

Epistemic (all)	n	Evaluative (n > 9)	n	Uptake (all)	n	Emotive (n > 9)	n	Descriptive (n > 9)	n	Ethical (n > 1)	n
*right*	1,477	*good*	512	*alright*	277	*amazing*	103	*weird*	89	*fair*	11
*true*	350	*nice*	199	*fine*	224	*funny*	79	*interesting*	66	*harsh*	5
*wrong*	11	*cool*	130	*okay*	96	*ridiculous*	51	*crazy*	57	*poor*	4
*correct*	7	*brilliant*	63	—	—	*awful*	34	*different*	16	*mean*	4
*impossible*	3	*great*	58	—	—	*horrible*	28	*strange*	16	*nasty*	3
*incorrect*	2	*lovely*	44	—	—	*disgusting*	26	*clever*	15	*naughty*	3
*exact*	1	*terrible*	39	—	—	*awesome*	24	*cute*	13	*unfair*	2
*definite*	1	*bad*	37	—	—	*hilarious*	23	*mental*	12	*scandalous*	2
*unlikely*	1	*incredible*	15	—	—	*annoying*	15	*pretty*	12	*generous*	2
—	—	*fantastic*	14	—	—	*sad*	12	*stupid*	12	*vile*	2
—	—	*perfect*	12	—	—	*exciting*	11	*beautiful*	11	—	—
—	—	—	—	—	—	—	—	*mad*	10	—	—
—	—	—	—	—	—	—	—	*easy*	10	—	—

### Most Frequent Adjectives Per Semantic Class

Table 2 lists the most frequent adjective types per semantic class. The frequency thresholds selected are provided in the header of the table. It should be noted that the class of “epistemic” adjectives is strongly dominated by *right* and, to a much lesser degree, *true*, while the other classes show a much less steeply declining frequency distribution.

### Distribution of Semantic Classes Across Social Variables

**FIGURE 2:**
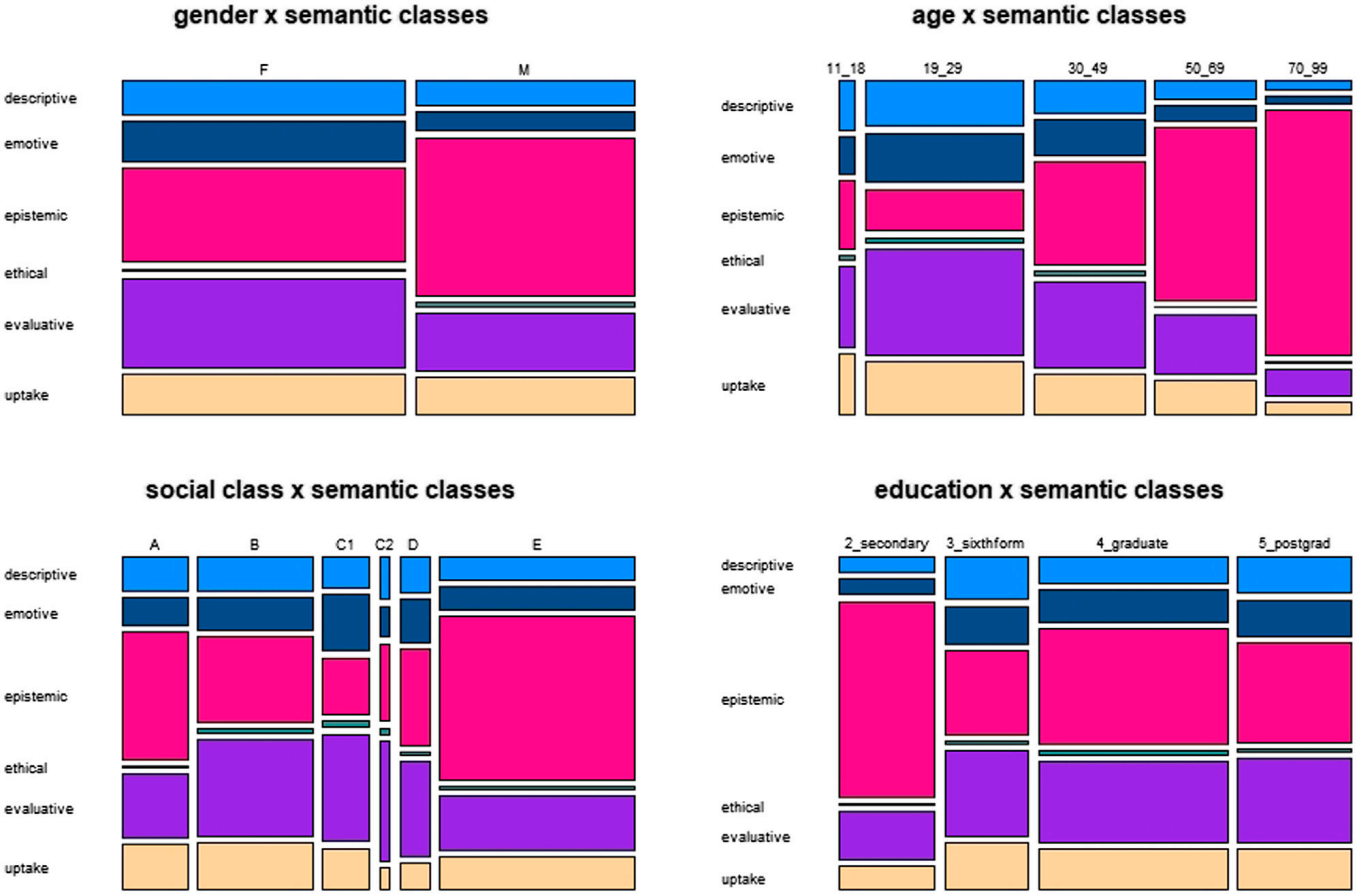
Distribution of semantic classes across social variables.

Figure 2 provides a survey of the frequency distribution of semantic classes across the four major social variables.

With regard to the variable gender, the proportions of the classes “ethical” and “uptake” are very similar for “female” and “male”. “Epistemic” adjectives account for a higher proportion of the tokens of men than of those of women, which is made up by higher proportions for “descriptive”, “emotive” and “evaluative” used by women.

Regarding age, we see very high proportions of “epistemic” adjectives for the age ranges 60 to 69 and 70 to 79. This corresponds to low proportions for the other classes in comparison to the younger age groups, who use adjectives of the other classes relatively more frequently.

The plot for the variable social class does not show a clear trend from class “A” to “E”. Instead, there is a U-shaped pattern with “C1” and “C2” in the center and similar trends in both directions: an increase of “epistemic” and a decrease of “evaluative” toward “A” and “B” as well as “D” and “E”.

The data for education also do not reflect a consistent trend, but instead seem to indicate more or less random variation.

### Distribution of the Twelve Most Frequent Adjectives across Social Variables

**FIGURE 3:**
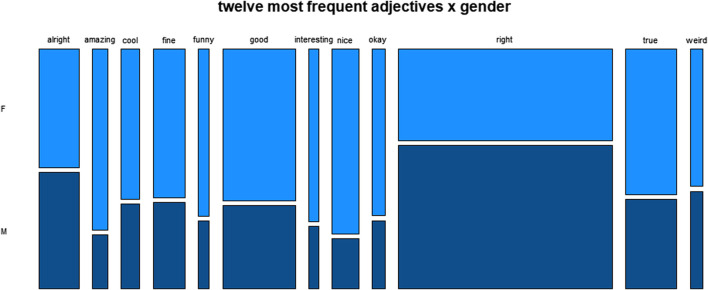
Distribution of twelve most frequent adjectives across gender.

**FIGURE 4:**
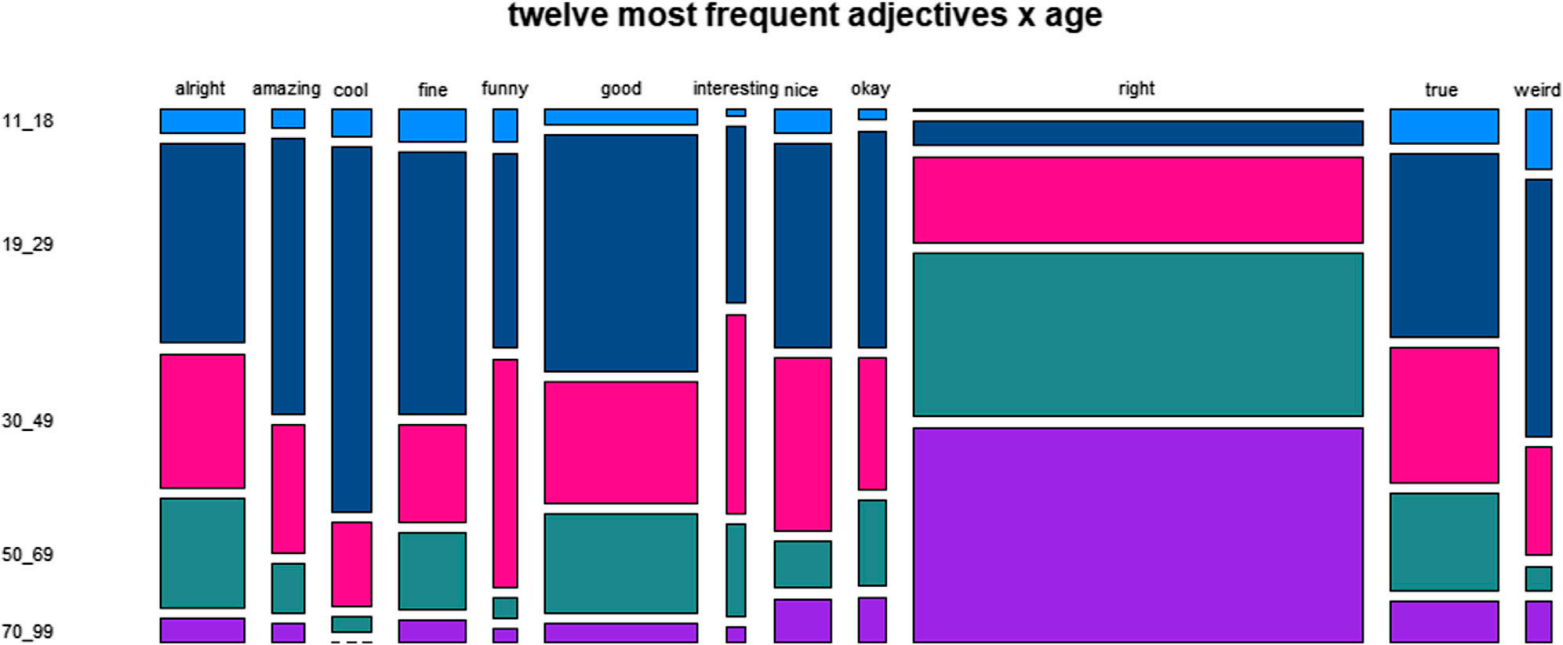
Distribution of twelve most frequent adjectives across age.

**FIGURE 5:**
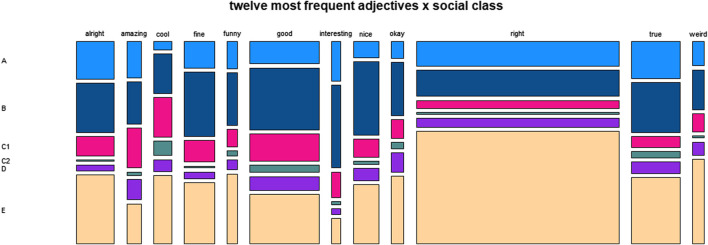
Distribution of twelve most frequent adjectives across social class.

**FIGURE 6:**
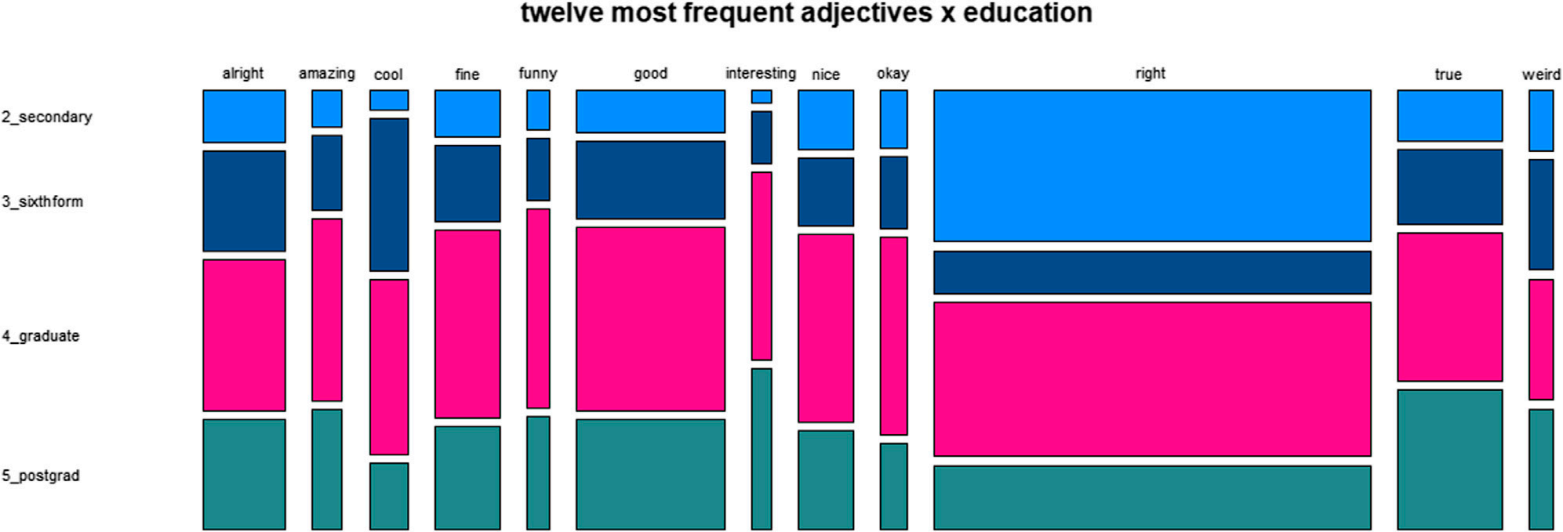
Distribution of twelve most frequent adjectives across education.

Figures 3-6 zoom in on the 12 most frequently used adjectives and represent their distribution across the four major social variables. Figure 3 representing the variable gender shows a more or less even distribution for the adjective *alright*, a strong male preponderance for *right*, and a female preponderance for the rest, which is particularly strong for the evaluative adjectives *amazing, funny, nice*.

Figure 4, rendering the data for the variable age, shows a general tendency for lower frequencies with higher age, in particular for *cool* and *weird*, and a reverse tendency for *right*.

As is indicated by Figure 5, the variable social class shows a clear trend for *right* to be used more frequently by members of higher social classes. Otherwise, there are no obvious tendencies. The same is true for the results regarding the variable education, as shown by Figure 6.

### Variation Across Conversations–Semantic Classes

**FIGURE 7:**
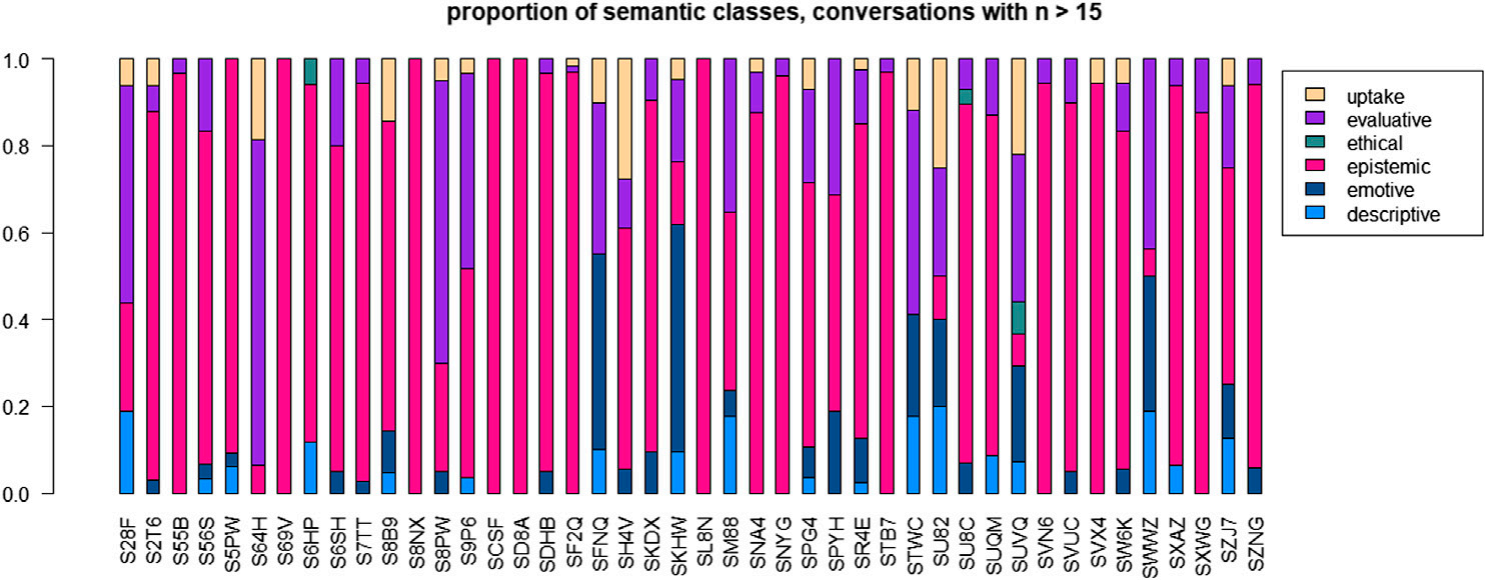
Distribution across semantic classes for 46 conversations with n > 15.

Social variation is compromised and superseded by situational variation (Labov 1966). Given the structure of our dataset, a good way of describing the effect of situational variation is to look at variation across conversations. Figure 7 represents the distribution regarding semantic classes in all 46 conversations which contain more than 15 instances of the target pattern. Overall, we notice a strong preponderance of the class “epistemic”, which is mainly caused by the very high frequency of *right*. However, some conversations show a more distributed pattern, e.g. conversations S28F, S9P6, SM88, STWC, SU82 or SWWZ. The conversations S28F, S64H, S8PW and STWC are dominated by the use of “evaluative” adjectives, the conversations SFNQ and SKHW by “emotive” adjectives. Assessing the interaction between situational variation and social variation will be left to the inferential statistics reported in *Inferential statistics and results*, because it is too complex for descriptive techniques.

### Individual Speaker Variation

Finally, we zoom in on differences between individual speakers, which are one of the main concerns of this paper. We will select different portions of the dataset, depending on how well they lend themselves to various ways of describing findings.

#### Speakers’ Choice of Semantic Classes

**FIGURE 8:**
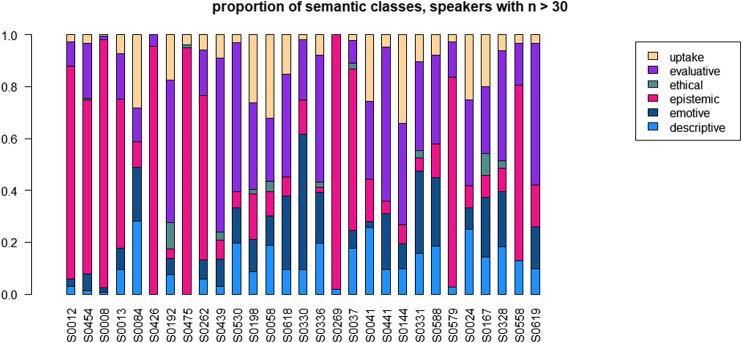
Distribution across semantic classes for 16 speakers with n > 50.

For the description of speakers’ choices of semantic classes, we have selected the data from the 30 speakers who boast a frequency of higher than 30 tokens. Figure 8 shows their distributions, ordered in terms of the frequency of uses of the pattern. Overall, the figure indicates a very large degree of inter-individual variation. The figure allows the following observations:

• a dominance of “epistemic” for 10 speakers: S0012, S0454, S0008, S0013, S0426, S0475, S0262, S0269, S0037, S0579;
• dominance for “evaluative” for eight speakers: S0192, S0439, S0530, S0618, S0336, S0441, S0328, S0619;
• dominance of “uptake” for two speakers: S0058, S0144;
• dominance of “emotive” for one speaker: S0330;
• and a quite balanced distribution for nine speakers: S0084, S0198, S0618, S0525, S0041, S0331, S0588, S0024, S0167

#### Speakers’ Choice of Specific Adjectives

**FIGURE 9:**
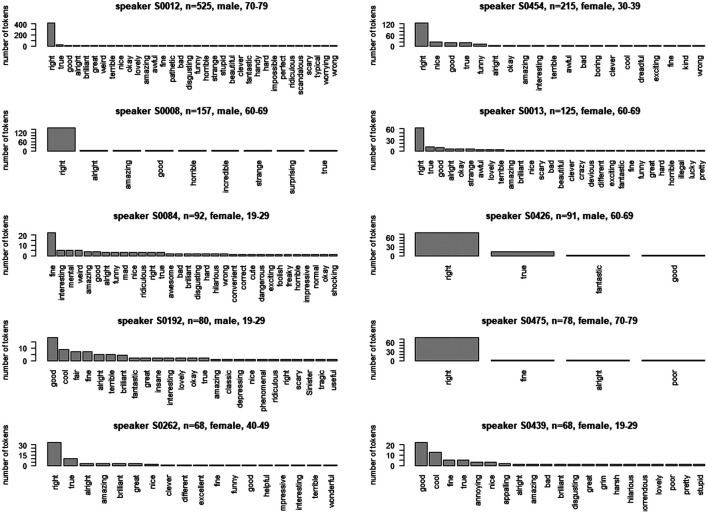
Distribution of adjectives for 10 speakers with highest frequencies of the pattern.

As representing the data for speakers’ choices of adjectives requires more space, we select the ten speakers with the largest number of tokens, from n = 525 to n = 68 (see Figure 9). Social characteristics are provided in the legend of all 10 panels of Figure 9. Not surprisingly, *right* turns out to be the dominant choice by far for as many as seven speakers (S0012, S0454, S0008, S0013, S0426, S0475, S0262). However, the degree of this dominance varies considerably from very extreme cases such as S0008 to more moderate ones such as S0454. What is also remarkable is that the slope of the curves outlined by the bars show very different shapes, reflecting the extent to which individual speakers favor only one or a small number of adjectives. In addition, it seems more or less impossible to correlate the differences between speakers with their social characteristics.

Figure 9 also provides the data for three speakers who do not have the routine of choosing *right* more frequently: S0084 favors the adjective *fine*, S0192 and S0439 the adjective *good*, followed by *cool* in both cases. Both of these speakers are young, as would be expected by the choice of *cool*, one is male and the other female.

Overall, the panels in Figure 9 show a mixture of speakers with extreme habits PLUS a range of other adjectives (S0454, S0013) and speakers with extreme habits WITHOUT noteworthy frequencies of other adjectives (S0008, S0475). This is an important observation that we will come back to in *Social and cognitive implications* below.

## Inferential Statistics and Results

### Aims

The aim of the inferential statistics reported in this section is to model the effects of social, situational and speaker variation. Specifically, we want to gauge.

- a. the effects of the four social variables gender, age, education and social class on the choice of semantic classes and the most frequent adjectives;
- b. the influence of situation-dependent variation by looking at the effects of the variable conversation;
- c. the influence of individual variation by looking at the variable speaker.

d. This will enable us to answer the question to what extent the variation found can be explained by social variables and to what extent it is superseded by situational and individual variation.

### Statistical Models

To reach these goals, we fitted mixed-effects binomial logistic regression models using gender, age, education and social class as fixed effects and speaker and conversation as random ones. This was done using the glmer function of the lme4 package (Version 1.1–23) in R (Version 4.0.2). The inclusion of the two random effects allows us to gauge the extent to which variation can be attributed to differences between individual speakers or conversations. The random effect speaker increases to the extent that individual speakers show a tendency to repeat their choices of semantic classes and adjectives, and the random effect conversation increases to the extent that choices are repeated by the participants within one conversation. It is in this way that the random effect speaker can be interpreted as an indicator of individual habits and inter-individual variation, and the random effect conversation as an indicator of same-speaker and other-speaker repetition in conversations.

The models targeted dependent variables on the two levels of analysis also used for the descriptive statistical analysis: the choice of semantic classes and the choice of specific adjectives. With regard to semantic classes, the binomial models compare the choice of one semantic class (e.g., “epistemic”) to all instances of all other classes. With regard to adjectives, we compare selected adjectives to all semantically similar adjectives from the same semantic class (see *Choice of Specific Adjectives* for more details). This corresponds to the conceptually plausible assumption that a speaker planning to use the pattern that’s Adj has a twofold paradigmatic choice between the general types of meaning they want to encode, on the one hand, and the specific adjective they want to use in order to do so, on the other.

### Results

Results will be reported in two steps: *Choice of semantic classes* deals with regression models targeting the choice of semantic classes, and *Choice of specific adjectives* with those targeting the choice of specific adjectives.

#### Choice of Semantic Classes

Mixed-effects regression models were fitted for the three semantic classes boasting the highest frequencies of tokens, i.e., “epistemic”, “evaluative” and “uptake”. In all cases, we fitted two models: one based on the full dataset and one in which all speakers who contributed only one token were excluded. Since the results of the two models were very similar, we will only report those based on the full dataset.

In Table 3, we present the summary of the regression model for the choice of the semantic class “epistemic”. The base categories are gender “female”, age “11 to 18”, social class “A” and education ‘secondary’. The summary indicates that the only social variable that was found to be significant was age, with speakers in the age ranges 50 to 69 and 70 to 99 showing a significantly incidence of using this semantic class. This is in line with expectations derived from the descriptive statistics reported in *Distribution of semantic classes across social variables*.

**TABLE 3:** Results from the mixed-effects binomial logistic regression model. The outcome variable was the use of an “epistemic” adjective. Random effects for conversation and speaker were included.

	Epistemic		
*Predictors*	*Log-Odds*	*CI*	*p*
(Intercept)	−1.87		
Gender [M]	−0.05	−0.48–0.37	0.801
Age [19_29]	−0.67	−1.62–0.28	0.166
Age [30_49]	−0.10	−1.11–0.91	0.848
Age [50_69]	1.22	0.27–2.16	0.012
Age [70_99]	2.92	1.84–4.01	<0.001
Social Class [B]	−0.58	−1.24–0.08	0.086
Social Class [C1]	−0.29	−1.08–0.50	0.468
Social Class [C2]	0.04	−1.36–1.44	0.952
Social Class [D]	0.42	−0.49–1.33	0.368
Social Class [E]	0.14	−0.53–0.81	0.685
Education [3_sixthform]	0.09	−0.64–0.82	0.807
Education [4_graduate]	0.38	−0.32–1.08	0.288
Education [5_postgrad]	0.58	−0.18–1.35	0.136
*Random Effects*			
σ^2^_conversation_	0.74	—	—
σ^2^_speaker_	1.37	—	—
ICC _conversation_	0.09	—	—
ICC _speaker_	0.33	—	—
*Number of observations*			
Observations	4,394	—	—
N _conversation_	931	—	—
N _speaker_	445	—	—

**TABLE 4:** Results from the mixed-effects binomial logistic regression model. The outcome variable was the use of an “evaluative” adjective. Random effects for conversation and speaker were included.

	Evaluative		
*Predictors*	*Log-Odds*	*CI*	*p*
(Intercept)	−0.87		
Gender [M]	−0.20	−0.50–0.10	0.196
Age [19_29]	0.03	−0.65–0.70	0.942
Age [30_49]	−0.04	−0.76–0.67	0.903
Age [50_69]	−0.36	−1.05–0.32	0.297
Age [70_99]	−1.55	−2.41–−0.69	<0.001
Social Class [B]	0.49	0.03–0.96	0.037
Social Class [C1]	0.41	−0.14–0.96	0.140
Social Class [C2]	0.77	−0.15–1.69	0.102
Social Class [D]	0.22	−0.42–0.86	0.502
Social Class [E]	−0.09	−0.57–0.40	0.730
Education [3_sixthform]	−0.28	−0.82–0.25	0.296
Education [4_graduate]	−0.25	−0.77–0.27	0.343
Education [5_postgrad]	−0.28	−1.70–−0.03	0.329
*Random Effects*			
σ^2^_conversation_	0.63	—	—
σ^2^_speaker_	0.80	—	—
ICC _conversation_	0.10	—	—
ICC _speaker_	0.15	—	—
*Number of observations*			
Observations	4,394	—	—
N _conversation_	931	—	—
N _speaker_	445	—	—

The two random effects conversation and speaker can be gleaned from the standard deviations reported in the summary, which are 0.74 and 1.37, respectively. Especially for the variable speaker, the score indicates a very strong effect of the repeated choices of individual speakers. A possible way of gauging the proportion of stochastic variation contributed by the random effects is to use the intra-class correlation coefficient (ICC; Nakagawa and Schielzeth 2010). This coefficient measures the correlation between the variance of a given random effect and the total variance. It is calculated by dividing the variance of a given random effect by the total random variation, i.e. the sum of the variance of all random effects and the variance of the logistic distribution. Since the latent-scale distribution-specific variance for the logit models we are using here is a constant given as π^2^/3 (Nakagawa and Schielzeth 2010), the ICC for the random variable speaker, for example, can be calculated as σ^2^_speaker_/(σ^2^_speaker_ + σ^2^_conversation_ + π^2^/3). The ICCs for the random effects speaker and conversation are 0.33 and 0.10, respectively. This can be interpreted as indicating that a proportion of 33% of the stochastic variation on the latent scale is contributed by the variable speaker, and 10% by conversation.

Table 4 reports the summary of the regression model for the class “evaluative”. The model indicates a weak but significant positive effect for social class “B” and a strong and highly significant negative effect for the age range 70–99. The variance rendered for each of the random effects are lower than for the class “epistemic”–0.40 for conversation and 0.64 for speaker. However, with an ICC of 15%, the contribution of the variable speaker to the stochastic variation remains considerable (ICC_conversation_ = 10%).

The regression model for the semantic class “uptake” reported in Table 5 indicates almost equally strong effects of the two random variables, with ICCs amounting to 14% for conversation and 15% for speaker. The only relevant social predictor is again age, with a weak but significant decrease associated with the age range 70–99.

**TABLE 5:** Results from the mixed-effects binomial logistic regression model. The outcome variable was the use of an “uptake” adjective. Random effects for conversation and speaker were included.

	Uptake		
*Predictors*	*Log-Odds*	*CI*	*p*
(Intercept)	−1.93		
Gender [M]	0.33	−0.01–0.68	0.056
Age [19_29]	0.38	−0.39–1.15	0.330
Age [30_49]	0.23	−0.60–1.06	0.586
Age [50_69]	−0.11	−0.89–0.67	0.778
Age [70_99]	−1.22	−2.18–0.26	0.013
Social Class [B]	0.21	−0.33–0.74	0.445
Social Class [C1]	−0.14	−0.79–0.50	0.669
Social Class [C2]	−1.10	−2.34–0.13	0.080
Social Class [D]	−0.66	−1.45–0.13	0.100
Social Class [E]	0.10	−0.46–0.66	0.725
Education [3_sixthform]	−0.15	−0.76–0.46	0.632
Education [4_graduate]	−0.36	−0.96–0.23	0.228
Education [5_postgrad]	−0.54	−1.19–0.12	0.108
*Random Effects*			
σ^2^_conversation_	0.82	—	—
σ^2^_speaker_	0.85	—	—
ICC _conversation_	0.14	—	—
ICC _speaker_	0.15	—	—
*Number of observations*			
Observations	4,394	—	—
N _conversation_	931	—	—
N _speaker_	445	—	—

In sum, the regression models suggest that effects of the fixed social variables on the choice of semantic classes are limited for all three semantic classes, while those of the random effects conversation and especially speaker are considerable throughout and very strong for the semantic class “epistemic”.

#### Choice of Specific Adjectives

The two top-ranking adjectives from the classes “epistemic”, “evaluative” and “uptake” were selected for regression models targeting the choice of specific adjectives: *right* and *true*, *good* and *nice*, and *alright* and *fine*, respectively. We fitted models that compared these adjectives to all quasi-synonymous adjectives of the same class. For example, *right* was compared to all other epistemic adjectives with the meaning “true, correct”, i.e., *true, correct, definite* and *exact*. *Good* was compared to the 18 other positive evaluative adjectives, including *brilliant, cool, excellent, fantastic, great* and *lovely*. This corresponds to the assumption that speakers select the adjectives from the pool of all those that can be used in the pattern in a given context. Since the group of uptaking adjectives includes no more than three adjectives, i.e., *alright, fine* and *okay*, the two adjectives *alright* and *fine* were compared to all other adjectives.

Rather than rendering the complete summaries of the regression models for all six adjectives, we restrict ourselves to reporting fixed effects that are significant at 5% level (stated as estimates and indicators of significance levels), random effects (stated as standard deviations) and ICCs per adjectives. This is summarized in Table 6.

**TABLE 6:** Results summary for mixed-effects logistic regression models for *right, true, good, nice, alright* and *fine.* Random effects for conversation and speaker were included.

Adjective	Significant fixed effects (estimate, significance level)	Random effects (standard deviation)	ICCs
*right*	Compared to all other epistemic adjectives meaning “true, correct”		
	Age [30_49]: 2.85*	Conversation: 1.22	15%
	Age [50_69]: 4.51***	Speaker: 2.24	51%
	Age [70_99]: 6.50***		
*true*	Compared to all other epistemic adjectives meaning “true, correct”		
	Age [30_49]: -3.44**	Conversation: 1.20	14%
	Age [50_69]: -4.88***	Speaker: 2.39	55%
	Age [70_99]: -6.74***		—
*good*	Compared to all other positive evaluative adjectives		
	—	Conversation: 0.41	4%
	—	Speaker: 0.66	11%
*nice*	Compared to all other positive evaluative adjectives		
	Gender [M]: -0.72*	Conversation: 0.85	16%
	—	Speaker: 0.74	12%
*alright*	Compared to all adjectives		
	Gender [M]: 0.69**	Conversation: 1.15	24%
	Age [70_99]: -1.42*	Speaker: 0.98	17%
	Social class [D]: -1.36*		
*fine*	Compared to all adjectives		
	Age [70_99]: -1.38*	Conversation: 0.84	13%
		Speaker: 1.15	25%

The two epistemic adjectives *right* and *true* show opposite trends regarding the variable age, with *right* being favored with increasing age and *true* being disfavored. These effects are huge. age is also a relevant variable for the choice of the two “uptake” adjectives *alright* and *fine*. “Male” gender has a reducing effect on the choice of *nice* and an increasing one on the choice of *all right*. social class “B” has a reducing effect on *right*, and social class “D” also a reducing one on *alright*. Overall, the amount of variation that can be explained with the help of fixed social variables is astonishingly low, except for age with respect to *right* and *true*. In contrast, as in the case of the choice of semantic class, the two random variables speaker and conversation show strong effects on the choice of adjectives. In all cases except *nice* and *alright*, the effect for speaker is much stronger than that for conversation. *Right* and *true* stand out with stunningly high ICC scores in addition to the large effects for age, which suggest that the dominant factors determining the choice of these two adjectives in the pattern are speakers' habits–observable within and across conversations–and self- and other-repetition in conversations.^5^

## Discussion

In this section we will first summarize the findings. These will then be discussed with regard to their social and cognitive implications. Finally, we will examine the relevance of these implications for the study of language variation and change. Throughout, we will take the perspective of the so-called *Entrenchment-and-Conventionalization Model* (Schmid 2020). We consider this model to be particularly suited for explaining the findings, because it integrates linguistic usage patterns, their conventionalization in the community and their entrenchment in the minds of individuals and tries to explain how the interaction between these three elements controls language structure, variation and change.

### Summary of Findings

Speakers can use a wide range of adjectives in the lexico-grammatical pattern that’s Adj in order to express various meanings (which can also be combined in specific utterances). We investigated 4,394 tokens of this pattern retrieved from BNC2014, originally produced by 445 speakers using 159 adjective types in 931 conversations. The descriptive and inferential statistics presented in this paper converge in the following findings:

• Speakers vary very strongly with regard to the frequency with which they a) use the pattern, b) encode the different meanings, and c) use the different adjectives.
• Overall, the effects of the social variables on the observed frequencies were fairly limited: higher age was found to have an increasing effect on the class of “epistemic” adjectives and the choice of the most frequent adjective *right*, and a decreasing effect on “evaluative” and “uptake” adjectives as well as the epistemic adjective *true.* Gender influenced the choice of *nice* and *alright*, social class the choice of the semantic class “evaluative” and the adjectives *right* and *alright.*
• The effect of situational variation–approached via the random variable conversation–was found to be high throughout.
• Confirming the results of the descriptive statistical analysis, individual variation–approached via the random variable speaker–was also found to have very strong effects on the choices of semantic classes and the adjectives focused on, with the class “epistemic” and the adjectives *right* and *true* standing out with extremely high effects of speaker repetition.

### Social and Cognitive Implications

From the social perspective of the speech community, the sequence that’s Adj qualifies as a highly conventionalized lexico-grammatical pattern whose use is motivated by a range of communicative functions. This means that among the members of the speech community, there is a mutually expected onomasiological regularity linking the goal to encode the various meanings of the pattern to its form and specific variants. Looking at the aggregated frequency distribution reported in *Distribution of tokens and types across semantic classes* and *Most frequent adjectives per semantic class*, one gets the impression that the pattern as such, its semantic variants and its specific instances such as *that’s right, that’s true* or *that’s good* are indeed widely agreed upon means of reaching recurrent communicative goals. This is basically what is meant when we call the pattern *conventional*.

In general, this impression is certainly correct, but the aggregated macro-perspective glosses over the considerable variation found regarding the frequencies of choices of semantic classes and specific adjectives in different conversations and by individual speakers. From this perspective, the behavior of the speakers in the corpus turns out to be all but uniform.

How can these findings be explained? With regard to situational variation, there is a range of well-established factors that are likely to cause the effects observed for conversation: classic situational factors such as participants, setting, activity type, topic and register readily come to mind here. These could easily be looked at in greater detail, because a lot of the information that is required is available in the BNC2014 metadata.

In addition, and from a more cognitive and psycholinguistic perspective, one can assume that the participants involved in a conversation show the well-known tendency to repeat identical or semantically similar tokens of the pattern. This tendency has been described in terms of concepts such as *accommodation* (Giles et al., 1991; Giles and Ogay 2007), *alignment* (Pickering and Garrod 2004), *co-adaptation* (Larsen-Freeman and Cameron 2008; Schmid 2020), *dialogic resonance* (Du Bois 2014), *priming* (Pickering and Ferreira 2008) or *persistence* (Bock 1986). These notions can be invoked to explain the strong effects of the random variable conversation, because the interpersonal and psychological tendencies they refer to are reflected in the repetition of semantic classes and specific adjectives in the course of individual conversations.

As far as individual variation is concerned, the results concerning speakers' choices of adjectives (reported in *Individual speaker variation*) and those concerning the random variable speaker in the mixed-effects regression models (see *Choice of specific adjectives*) indicate two things: first, that many speakers have routinized habits of using specific patterns such as *that’s right*, *that’s true* or *that’s fine*; and second, that speakers’ habits differ considerably and in ways that are not, or only weakly, determined by their social characteristics. It is true that in *Speakers’ choice of specific adjectives* we found that many speakers showed a strong preference for the pattern *that’s right*. But it is equally true that others hardly ever used this pattern and instead showed a high proportion of uses of *that’s true* or *that’s fine* or *that’s good* in their data.

These findings can be interpreted from a cognitive perspective, if one accepts the logic of frequency-driven entrenchment (Langacker 1987; Schmid 2007). The premise of this logic is that what has become more entrenched by frequent repetition is activated more effortlessly and more quickly than what is less entrenched due to less frequent processing (Schmid 2017a; Schmid 2017b). If this premise is correct and if we reverse the perspective, one can assume that if a linguistic element or pattern is produced by a given speaker more frequently than another one which competes to encode the same information (Geeraerts 2017), then this element or pattern is more strongly entrenched in the mind of this speaker than the competing elements relative to the communicative task at hand (Schmid 2020). For example, one would assume that the pattern *that’s right* is strongly entrenched in the mind of speaker S0012, who uses this pattern as many as 407 times in the BNC2014 data, with the quasi-synonymous pattern *that’s true* trailing behind in second rank with as few as 21 instances (see Figure 9 in *Speakers’ choice of specific adjectives*). In contrast, speaker S0084 seems to have a particularly strongly entrenched representation of the pattern *that’s fine*, and speaker S0439 of the pattern *that’s good*.

It is tempting to claim that these strongly entrenched specific patterns are represented as holistic chunks in the minds of the respective speakers (Sinclair 1991; Wray 2002; Nelson 2018). Rather than putting together *that’s* and *right* or *that’s* and *fine* compositionally by means of syntactic operations, speakers who routinely use these patterns probably have them available as ready-made chunks or prefabs in their mental lexicons (Gobet et al., 2001; Ellis 2017). However, it is unclear how many repetitions are required to create such a chunk in the mental lexicon, and also, from a methodological point of view, how many attestations would be required as evidence for the existence of such a chunk (Blumenthal-Dramé 2012; Blumenthal-Dramé 2017). Therefore, following the arguments put forward by Schmid (2020), we argue that the chunk-like processing and representation of sequences is best accounted for in terms of particularly strong syntagmatic associations giving rise to a very high sequential predictability. In this way, more or less frequent patterns do not have to be forced into a categorical distinction between “chunk” and “compositional sequence”, but can instead be described on a scale of strength of syntagmatic associations, from extremely strong and therefore essentially chunk-like to somewhat looser, as in the case of collocations or complementation patterns. The strength of syntagmatic associations is not only determined by the frequency of earlier processing episodes, but also by symbolic, paradigmatic and pragmatic associations. Symbolic associations connect the forms of the pattern to the various meanings. Paradigmatic associations connect the competitors in a given variable slot (e.g. *right* and *true* in the adjective slot of the pattern). Pragmatic associations connect the forms to communicative motivations and goals such as “express approval” or “express uptake” (Schmid 2014). From this perspective, the use of the pattern and its specific variants is not modeled as an either holistic or compositional access-retrieve-combine operation, but instead as the incremental activation of a dynamic pattern of the four types of association. In line with theories of predictive coding (Friston 2010; Huang and Rao 2011; Kroczek and Gunter 2017), this model of processing links representations based on prior experience with processing based on current perception and context.

An additional advantage of this associative approach is that it provides an integrated perspective on interesting differences between the “usage profiles” (Schmid and Mantlik 2015) of different speakers (see again *Speakers’ choice of specific adjectives*). Speaker S0008, for example, is a very extreme case: the 157 tokens of the variable pattern that’s adj that he contributes to the corpus are divided into as many as 149 tokens of *that’s right* and only one token each of *alright, amazing, good, horrible, incredible, strange, surprising* and *true*. The highly routinized repetition of *that’s right* can be modeled as being triggered by an associative complex connecting the communicative goal of expressing consent and approval by means of the sequence *that’s right*. This sequence seems to be so strongly entrenched pragmatically, symbolically and syntagmatically in the mind of this speaker that it does not seem to have any serious paradigmatic competitors for reaching the given communicative goal. This is presumably different in the case of speaker S0454, who also has a large proportion of uses of *that’s right* (n = 124), but contributes another 91 tokens, among them 22 tokens of *nice*, 19 of *true* and *good* and nine of *funny*. This distribution can be interpreted as reflecting the co-existence of a strong specific representation of the syntagmatic sequence *that’s right* (which is strongly triggered by pragmatic associations) and an entrenched variable pattern which is also connected to the function “evaluative” in addition to “epistemic”. Further illuminating examples are speakers S0084 and S0192, whose use of the pattern is apparently dominated by several pragmatic motivations, including “uptake” and “evaluative”, as is indicated by the frequent use of the adjectives *fine* as well as *interesting, mental, weird, amazing* and *good* in the case of S0084 and *good, cool, fair, fine, alright, brilliant* and *terrible* in the case of S0192.

Translating these claims into the more established but also more rigid and less dynamic framework of Construction Grammar (Goldberg 1995; Goldberg 2006; Goldberg 2019; Hilpert 2014)–which has been the elephant in the room anyway –, we can say that speakers differ with regard to how strongly the highly schematic that’s adj construction or lower-level schemas like “epistemic” or “evaluative” or certain lexically-specific constructions such as *that’s right* or *that’s fine* are represented in their constructicons.

In sum, we have claimed that underneath the apparent uniform linguistic behavior on the aggregate macro-level of the community we find significant differences in the frequencies of usage patterns, and that these differences can be interpreted as indicating a considerable degree of covert “speaker-specific cognitive variation” (Schmid 2020: 308). In the next, penultimate section we will discuss the ways in which individual differences and covert cognitive variation can affect variation and change on the macro-level and why they should be of interest to sociolinguists and students of language change.

### Implications for the Study of Variation and Change

#### Variation

In sociolinguistics and language change, language has traditionally been framed as an “object possessing orderly heterogeneity” (Weinreich et al., 1968: 100), with *orderly* essentially referring to a differentiation of the behavior of groups of speakers which is systematic in the sense that it can be correlated with social and situational factors. Associated with the variables gender, age, education and social class, in the present case study this orderly type of variation turned out to be less dominant than individual variation, which is unorderly by definition. Individual differences turned out to contribute much more to the overall variation observed than linguists on the hunt for orderly heterogeneity are usually happy to see.

One way out of this dilemma would be to state that the frequency distributions found are no more than what we have described them as, i.e., expectable effects of cognitive processes like entrenchment and priming. As such, one could conclude, they do not have anything interesting to contribute to our understanding of social variation and language change. However, this might be too easy a way out. After all, it is generally assumed in quantitative sociolinguistics and historical linguistics that frequency distributions reflect and reinforce sociolinguistic patterns and that differences in usage frequencies can trigger and index language change. This would suggest that individual frequency differences should not be ignored in the study of social variation and language change, but instead by related to social and situational variation.

How can this be achieved? What are the links between individual and social variation? In our view, individual variation neither compromises nor supersedes social variation, but rather generally subserves it. The fundamental assumption underlying sociolinguistics is that the entrenched routines and habits of speakers on all levels of language–from phonology and morphosyntax to pragmatics–are influenced by social factors or at least correlate with them. These social factors include the usual suspects, for example frequency of social interaction, the structure and density of social networks and communities of practice, people’s tendency to seek solidarity and signal distance and to identify and align with members of their social groups and networks. Both orderly social and seemingly random individual variation are ultimately based on the routines and habits of speakers. Variation is considered to be orderly to the extent that these routines and the differences between them are correlated with some aspects of social structure or situated social interaction. In the *Entrenchment-and-Conventionalization Model* (Schmid 2020), it is generally assumed that speakers' patterns of social interactions and their social identities ultimately do shape the associative networks in their minds, because they determine the linguistic experiences that speakers accumulate. However, there seems to be a considerable residue of individual habits and whims which mainly have a cognitive foundation in the repetition-driven routinization of past behavior. It would therefore not be surprising if a closer look at existing quantitative sociolinguistic studies revealed that in many cases the usage patterns of individual speakers were a central source of variation to be taken much more seriously.

#### Change

The claim that individual variation should attract more attention gains further weight when we consider that the behavior of individual speakers can trigger and support various types of language change. The most obvious way in which this can happen is the use and subsequent repetition of new fillers of variable slots of existing patterns (Schmid 2020: 137). For example, tracing back the use of that’s adj and that is adj in the Early English Books Online corpus (Petré 2016), one finds that for a considerable time after the pattern seems to have been borrowed from French around 1,500, only the epistemic adjectives *true* and *false* were used, with *right* not appearing before the middle of the 17th century. Descriptive and evaluative adjectives such as *good, excellent* or *strange* entered the scene around Shakespeare’s time. These innovations must have been introduced by individual speakers, and their propagation was presumably supported by repeated use by a small number of speakers to begin with. Concrete illustrations of how this works in the case of other patterns can be found in Schmid and Mantlik (2015) and Mantlik and Schmid (2018).

High frequencies of repetition of specific sequences such as *that’s right* by individual speakers also have the potential to trigger and support macro-changes like pragmaticalization (Diewald 2011) and grammaticalization (see Schmid 2020: Ch. 19 for discussion). In fact, *that’s right* can be considered a case in point if one argues that–especially for those speakers who repeat this sequence very frequently–it is no longer an expression of epistemic stance, signaling a truth-related token of agreement, but has turned into a generalized discourse marker essentially on a par with the “uptake” adjectives *alright, fine* and *okay*. *Fine*, too, can be claimed to have undergone a similar pragmaticalization process from expressing an evaluative and hence propositional meaning to mainly serving a discursive function. Recent studies on the contribution of individual differences in language change–e.g. by Schmid and Mantlik (2015); Baxter and Croft (2016); Petré and Van de Velde (2018); Anthonissen (2020a); Anthonissen (2020b); Fonteyn and Nini (2020); Petré and Anthonissen (2020)–are accumulating more and more evidence suggesting that especially the early phases of the propagation of innovations are marked by massive variation among speakers, with some using a new element or pattern highly frequently while many contemporary writers do not use it at all (Schmid 2020: 320).

## Conclusion

The explicit mission of quantitative variationist sociolinguistics has been–and will continue to be–to unveil sociolinguistic patterns, i.e., to identify correlations of types of linguistic behavior with types of speakers and types of situations. Individual differences have been considered an unwelcome, uninteresting and largely uncontrollable source of variation in this endeavor. Therefore, with notable exceptions (see e.g., Tagliamonte and Baayen 2012), researchers in this field have tended not to pay much attention to the effect of individual variation, even if speakers or test participants were included as random effects in mixed-effects models or random forests. Against this backdrop, the main thrust of this paper is of a theoretical and methodological nature, rather than related to the content in terms of subject-matter. We have argued that the study of individual variation should complement the study of social (including regional and situational) variation, mainly because individual variation ultimately subserves social variation and because it plays an important role in language change. The suggestions we have made as to how the study of individual differences can be approached are just a starting-point. They are meant to encourage scholars working in quantitative and especially computational sociolinguistics to step up their efforts to take individual variation on board in future work and to develop more sophisticated tools and techniques for investigating it.

## Data Availability Statement

Publicly available datasets retrieved from http://corpora.lancs.ac.uk/bnc2014/ were analyzed in this study. The code used for processing the data can be found at https://github.com/wuqui/IndVar. The dataset and R-Code used for the regression models can be found in the Supplementary Material.

## Author Contributions

H-JS developed the conception and design of the study; QW conducted the data retrieval and processing; HK and SF developed the conception of the statistical analyses and SF performed them; H-JS wrote the first draft of the manuscript; QW wrote the first draft of *Data source* and *Data retrieval*; all authors contributed to manuscript revision and read and approved the submitted version.

## Conflict of Interest

The authors declare that the research was conducted in the absence of any commercial or financial relationships that could be construed as a potential conflict of interest.

## Footnotes

1
http://corpora.lancs.ac.uk/bnc2014/

2
http://ucrel.lancs.ac.uk/usas/

3
https://cqpweb.lancs.ac.uk/

4
Desagulier (2014) provides an R-script for extracting linguistic data and social metadata from the offline version of the BNC2014. The script allows users to perform basic queries using word and part-of-speech information and includes some metadata about speakers in its output. It also offers user-friendly options to create some exploratory plots and to export results to text files. However, the script does not provide options to formulate more complex queries, e.g. filtering based on position and tag sequences, or to retrieve and export semantic information and metadata about conversations and speakers required for this study.

5 Recalling the quite extreme preference of speakers S0012, S0454 and S0008 reported for *right* in *Speakers’ choice of specific adjectives*, one might assume that these three speakers are mainly responsible for the effects of age and speaker. Therefore we also fitted models in which these three speakers were excluded, but the effects of age and speaker remained almost equally large.
